# Research on an Intelligent Lightweight-Assisted Pterygium Diagnosis Model Based on Anterior Segment Images

**DOI:** 10.1155/2021/7651462

**Published:** 2021-07-29

**Authors:** Bo Zheng, Yunfang Liu, Kai He, Maonian Wu, Ling Jin, Qin Jiang, Shaojun Zhu, Xiulan Hao, Chenghu Wang, Weihua Yang

**Affiliations:** ^1^School of Information Engineering, Huzhou University, Huzhou 313000, China; ^2^Zhejiang Province Key Laboratory of Smart Management & Application of Modern Agricultural Resources, Huzhou University, Huzhou 313000, China; ^3^The First People's Hospital of Huzhou, Huzhou313000, China; ^4^The Affiliated Eye Hospital of Nanjing Medical University, Nanjing 210029, China

## Abstract

**Aims:**

The lack of primary ophthalmologists in China results in the inability of basic-level hospitals to diagnose pterygium patients. To solve this problem, an intelligent-assisted lightweight pterygium diagnosis model based on anterior segment images is proposed in this study.

**Methods:**

Pterygium is a common and frequently occurring disease in ophthalmology, and fibrous tissue hyperplasia is both a diagnostic biomarker and a surgical biomarker. The model diagnosed pterygium based on biomarkers of pterygium. First, a total of 436 anterior segment images were collected; then, two intelligent-assisted lightweight pterygium diagnosis models (MobileNet 1 and MobileNet 2) based on raw data and augmented data were trained via transfer learning. The results of the lightweight models were compared with the clinical results. The classic models (AlexNet, VGG16 and ResNet18) were also used for training and testing, and their results were compared with the lightweight models. A total of 188 anterior segment images were used for testing. Sensitivity, specificity, F1-score, accuracy, kappa, area under the concentration-time curve (AUC), 95% CI, size, and parameters are the evaluation indicators in this study.

**Results:**

There are 188 anterior segment images that were used for testing the five intelligent-assisted pterygium diagnosis models. The overall evaluation index for the MobileNet2 model was the best. The sensitivity, specificity, F1-score, and AUC of the MobileNet2 model for the normal anterior segment image diagnosis were 96.72%, 98.43%, 96.72%, and 0976, respectively; for the pterygium observation period anterior segment image diagnosis, the sensitivity, specificity, F1-score, and AUC were 83.7%, 90.48%, 82.54%, and 0.872, respectively; for the surgery period anterior segment image diagnosis, the sensitivity, specificity, F1-score, and AUC were 84.62%, 93.50%, 85.94%, and 0.891, respectively. The kappa value of the MobileNet2 model was 77.64%, the accuracy was 85.11%, the model size was 13.5 M, and the parameter size was 4.2 M.

**Conclusion:**

This study used deep learning methods to propose a three-category intelligent lightweight-assisted pterygium diagnosis model. The developed model can be used to screen patients for pterygium problems initially, provide reasonable suggestions, and provide timely referrals. It can help primary doctors improve pterygium diagnoses, confer social benefits, and lay the foundation for future models to be embedded in mobile devices.

## 1. Introduction

Pterygium is a common and frequently occurring disease in ophthalmology. It is the degeneration and growth of conjunctival fibrovascular tissue on the cornea, which usually leads to astigmatism and dry eyes. Covering the pupil area can cause a significant decrease in vision. The main treatment is surgical resection [1]. Pterygium can usually be diagnosed by anterior segment images [1]. Anterior segment images are taken with a slit lamp digital microscope and obtained by the diffuse illumination method at a magnification of 10x. Professional ophthalmologists often diagnose ocular surface diseases by viewing the anterior segment images. At present, the incidence of pterygium disease in China is 9.84% [2], and there are few professional ophthalmologists in county-level and lower hospitals and community hospitals and other basic-level hospitals. Therefore, it is difficult for the basic-level hospitals to meet the needs of the huge number of pterygium patients. The problem was to be solved; an intelligent lightweight-assisted diagnosis model based on anterior segment images is proposed in the study. The model can help nonprofessional ophthalmologists in the primary hospital to make preliminary diagnoses of pterygium patients and help them obtain pterygium grading (three types of normal, observation, and surgery) to get an accurate referral. The model can also be embedded in mobile phones to assist users in self-screening. Some primary doctors may not provide effective services to pterygium patients, and this model can help to solve the problem.

The combination of ophthalmology and artificial intelligence (AI) has become closer with the development of AI [3–9]. In 2016, a deep learning model was proposed by the Google team; it can diagnose DR automatically through fundus images [10]. Deep learning models have been used by lots of researchers to diagnose DR [11–14] since then. In addition to DR, researchers have used deep learning methods to detect common fundus diseases, including glaucoma [15–17], retinal vein occlusion [18, 19], age-related macular degeneration [20–22], and even research on the classification of multiple common fundus diseases [23]. These studies obtained good results.

There are many studies on the diagnosis of fundus-related diseases using deep learning methods but relatively few studies on ocular surface diseases. Pterygium is a common disease on the ocular surface. AI research on pterygium is mainly pterygium detection. Traditional learning methods mainly extract the pterygium characteristics in anterior segment images to detect pterygium. Related researchers have used the adaptive nonlinear enhancement method, SVM, to segment pterygium tissue to detect pterygium [24–26]. In recent years, researchers such as Mohd Asyraf Zulkifley have used neural networks, DeepLab V2, and other deep learning methods to detect and segment pterygium [27–29]; researchers such as Zamani et al. have used a variety of deep learning models to perform two-class detection of pterygium [30]. Existing research on pterygium detection is mostly based on the two-class detection of pterygium based on anterior segment images. It has not been further determined whether the pterygium is operated on, which cannot meet the needs of precision medicine. The deep learning models used for detecting pterygium are currently mostly classic; the excessive number of parameters requires considerable space and cannot be used on mobile terminals or low-configuration devices.

An intelligent lightweight-assisted pterygium diagnosis model is designed by using transfer learning in this study. The model detects normal images, pterygium observation periods, and pterygium surgery periods from anterior segment images. Comparative research with classic models is carried out simultaneously and reported as follows.

## 2. Materials and Methods

### 2.1. Data Source

The Affiliated Eye Hospital of Nanjing Medical University provided anterior segment images for this study. The images were obtained from two models of slit lamp digital microscopy. In this study, 436 anterior segment images were used to train an intelligent lightweight-assisted pterygium diagnosis model. The dataset consists of 142, 144, and 150 anterior segment images for the normal, pterygium observation period, and pterygium surgery period, respectively. There were 188 images for model testing, which consisted of 61, 62, and 65 anterior segment images for the normal condition, pterygium observation period, and pterygium surgery period, respectively. The patients' gender and age were not restricted when selecting the images. The personal information of patient-related was all removed from the images to avoid infringing on patient privacy.

**Figure 1:**
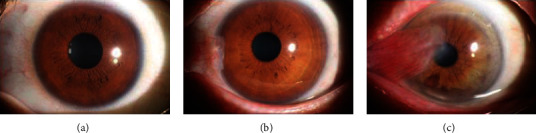
Three types of anterior segment images.

In this study, the images selected had high quality. Thus, ophthalmologists can easily diagnose whether the image shows a normal anterior segment or pterygium. The anterior segment images were either normal or showed pterygium. The image selected was diagnosed only as normal, pterygium observation period, or pterygium surgery period. The marking standard was [31] as follows. The normal anterior segment image is characterized by no obvious conjunctival hyperemia or proliferation, and the cornea is transparent; the anterior segment image of the pterygium observation period is characterized by the horizontal length of the pterygium head tissue invading the limbus of the cornea <3 mm; the anterior segment image of the pterygium surgery period is characterized by the horizontal length of the pterygium head tissue invading the limbus of the cornea ≥3 mm. The three types of anterior segment images were shown in Figure 1. There were two professional ophthalmologists who diagnosed the anterior segment images independently. If the diagnostic results of two ophthalmologists were identical, the final clinical diagnosis result was achieved. If the diagnostic results of two ophthalmologists were different, the final clinical diagnostic result was given by an expert ophthalmologist.

Figure 1(a) is the normal anterior segment image; Figure 1(b) is the pterygium observation period anterior segment image; Figure 1(c) is the pterygium surgery period anterior segment image.

### 2.2. Data Augmentation

**Figure 2:**
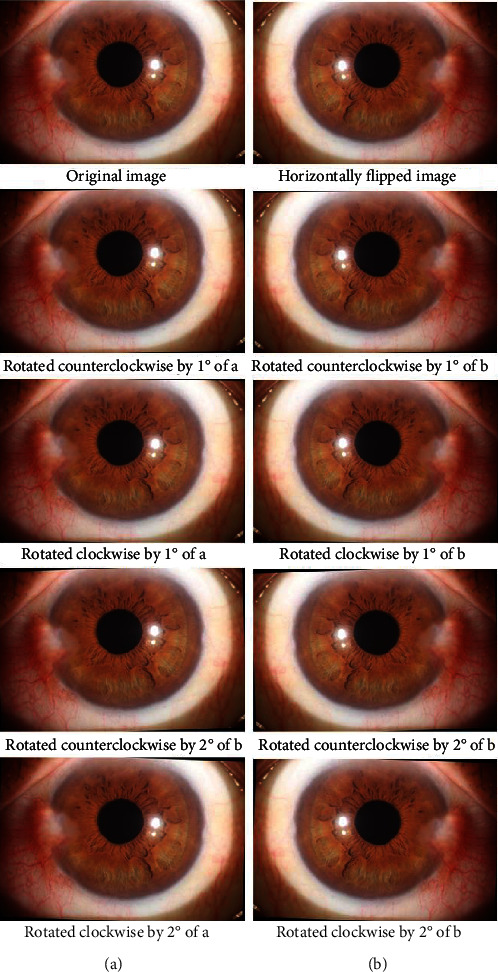
Original image and its augmented images.

The quantity of training data was too small. Therefore, the original images were augmented by flipping and rotating the original image. First, the original image was flipped horizontally, and then, the original image and the horizontally flipped image were rotated clockwise and counterclockwise by 1° and 2°, respectively, so that the augmented image maintained the original medical characteristics. An original image and its augmented image are shown in Figure 2.

### 2.3. Lightweight Model Training

The study uses a MobileNet [32] model; the initial parameters used in the model were pretrained on the ImageNet Large Scale Visual Recognition Challenge (ILSVRC) [33] dataset. A total of 436 original anterior segment images and 4,360 augmented anterior segment images were used to train two intelligent lightweight assisted diagnosis models for detecting pterygium grading. The network structure is not changed, and only the final output is changed to 3 categories in the transfer learning process.

**Figure 3:**
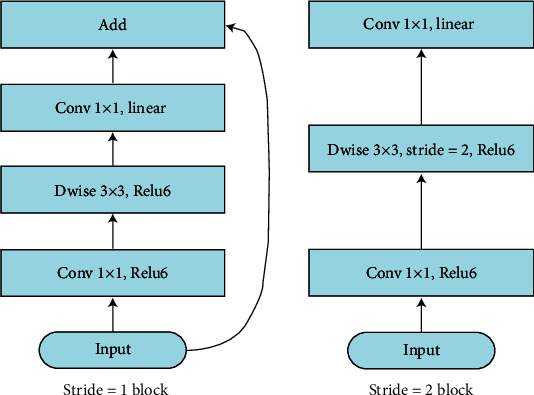
Bottleneck structure.

MobileNet is a lightweight model designed specifically for mobile and embedded terminals. This study focuses on the transfer learning of the MobileNet model with an inverted residual structure. Its basic network structure mainly includes convolutional layers, bottleneck layers, and an average pooling layer. The structure of the bottleneck layers is shown in Figure 3 [32], it usually includes pointwise convolution and depthwise convolution; when the stride is 1, the input is added to the output. The structure of MobileNet 2 is shown in [32].

In this study, a total of 436 original anterior segment images and 4,360 augmented anterior segment images were selected to train the two lightweight models. The images input to the two lightweight models were 224 × 224. Intelligent lightweight-assisted diagnosis models were obtained after training.

### 2.4. Classic Model Training

AlexNet [34], VGG16 [35], and ResNet18 [36] are three classic deep learning classification models. This study used 4,360 augmented anterior segment images to train three intelligent classic-assisted diagnosis models for detecting pterygium grading. The network structure of the three models with their initial parameters pretrained on the ILSVRC [33] dataset was used. The network structure is not changed, and only the final output is changed to 3 categories in the transfer learning process. The images input to the three models were 224 × 224. Intelligent-assisted diagnosis models were obtained after training. The results of the three models were compared to the lightweight models.

The server was used to train and test the five models. A computer was also used to test the five models because the basic hospitals usually do not have servers. The hardware configuration of the server used in this study is Intel (R) Xeon (R) Gold 5118 CPU, the main frequency is 2.3 GHz, the graphics card is Tesla V100, the video memory is 32 GB, and the operating system is Ubuntu 18.04. The hardware configuration of the computer used in this study is Intel (R) Core (TM) i5-4200M CPU, the main frequency is 2.5, the augmentation method is GHz, NVIDIA GeForce GT 720M x, the video memory is 1 GB, and the operating system is windows10.

### 2.5. Statistical Analysis

The SPSS 22.0 statistical software was used to analyze the results. The accuracy, size and parameters of models, time, sensitivity, specificity, F1-score, and AUC for the pterygium diagnostic models were calculated for the normal anterior segment image, pterygium observation period anterior segment image, and surgery period anterior segment image; then, ROC curves were plotted. The consistency between the expert and the model was evaluated by kappa value.

## 3. Results

A total of 188 anterior segment images were used to test the intelligent lightweight-assisted pterygium diagnosis models based on original data (MobileNet 1) and augmented data (MobileNet 2) for pterygium. The expert diagnosed 61 images as normal anterior segment, 62 as pterygium observation period, and 65 as pterygium surgery period. MobileNet 1 diagnosed 64 images as normal anterior segment, 55 as pterygium observation period, and 69 as pterygium surgery period. MobileNet 2 diagnosed 61 images as normal anterior segment, 64 as pterygium observation period, and 63 as pterygium surgery period. The two models' diagnostic results are shown in Tables 1 and 2.

A total of 188 anterior segment images were used to test the three classic intelligent-assisted pterygium diagnosis models (AlexNet, VGG16, ResNet18) based on augmented data (MobileNet 2) for pterygium. The three models' diagnostic results were compared with MobileNet 1 and MobileNet 2. Compared with the results of expert diagnosed, except for ResNet18, the sensitivity of the other four models for diagnosing the anterior segment image as normal was above 90%, the sensitivity of diagnosing the anterior segment image as pterygium observation period was up to 83.87% (AlexNet and MobileNet2), and the highest sensitivity of diagnosing the anterior segment image as pterygium surgery period was 86.15% (MobileNet1). The specificities of the five models for diagnosing anterior segment images as normal, pterygium observation period, and pterygium surgery period were mostly above 85%. Among them, the specificities of MobileNet 2 for diagnosing anterior segment images as the three grades were all above 90%, which showed that the models' misdiagnosis rates were low. The AUC values of MobileNet 2 for diagnosing anterior segment images as normal, pterygium observation period, and pterygium surgery period were the highest among the five models, which were 0.976, 0.872, and 0.891, respectively. The five models' evaluation results are compared in Table 3.

**Figure 4:**
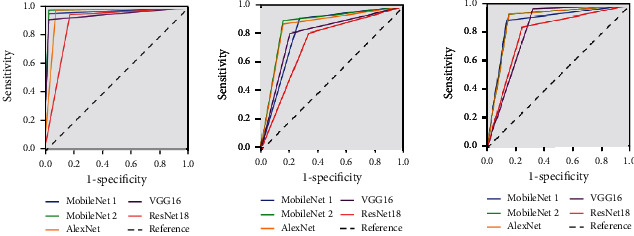
ROC of the five models for normal, pterygium observation period, and surgery period.

In Table 3, the sizes and parameters of the five models are compared. MobileNet 1 and MobileNet 2 had the smallest sizes and parameters among them, 13.5 M and 4.2 M, respectively. VGG16 had the largest size and parameters, 527 M and 138 M, respectively. As shown in Table 3, MobileNet 2 had the smallest space and the least number of parameters, and the evaluation indicators, such as sensitivity, specificity, F1-score, AUC, kappa value, and accuracy rate, still had good results. The AUC, kappa value, accuracy, and test time were the best among the 5 models. The five models' ROC curves of diagnosing the anterior segment image as normal, pterygium observation period, and pterygium surgery period are compared in Figure 4.

Time-S means the time of testing one image based on server; time-C means the time of testing one image based on the computer.

## 4. Discussion

Pterygium is a common ocular surface disease that can cause vision loss and affect appearance. It has a higher incidence among people working outdoors in rural and remote areas (such as fishermen and farmers). For vast rural and remote areas that lack professional medical resources for ophthalmology, the intelligent-assisted diagnosis model can provide a convenient method for screening pterygium for local patients. It can avoid the rush of patients to go to county hospitals or prefectural hospitals and reduce their financial burden. Additionally, the model further provides treatment suggestions to facilitate the referral of patients in need of surgery at the basic-level hospital; it can also reasonably allocate medical resources.

In 2012, the AlexNet model [34] won the championship of the classification in the ILSVRC competition. The network structure of the AlexNet model has 7 layers, while the network structure of the VGG model [35] has up to 19 layers and researchers often use VGG 16, which has 16 layers. The ResNet model [36] has up to 152 layers, but researchers often use ResNet 18 and ResNet 50. The network structures of the models almost are deep, so it is suitable for the extraction of more complex image features. The above models are classic and occupy a large amount of space and have many parameters. The MobileNet model is a lightweight model. The depth of the separable convolution kernel and linear bottleneck was used to reduce the number of parameters so that the model occupies a small space. The complexity of the anterior segment image is relatively low, so AlexNet obtained better results than the three classic models. MobileNet is further simplified based on the classic model and obtained the best diagnosis results among these models.

MobileNet2 had the best overall diagnosis results among the 5 models, but its sensitivity for diagnosing the pterygium observation period and pterygium surgery period was only 83.87% and 84.62%, respectively. The sensitivity was low because the number of the training samples was only 436. Although the images were augmented by 10 times, the augmented images were more similar to the original image. The effect improved, but the improvement was small.

As shown in Table 2, the MobileNet 2 model did not diagnose the pterygium surgery period images as normal images. Only pterygium observation periods were diagnosed as normal images. Most of the other errors were diagnosed between the pterygium observation period and pterygium surgery period. Patients diagnosed during the pterygium observation period and pterygium surgery period were recommended to go to the superior hospital for further confirmation, and the correct diagnosis was obtained after referral. Since the pterygium observation period images were diagnosed as normal anterior segment images, the model asked the user to diagnose again after obtaining the diagnosis result of the normal anterior segment image or asked the user whether to upload the image to the doctor. If the user had doubts about the diagnosis result, the user could upload the image, and a doctor confirmed the diagnosis result.

In this study, the parameters of the MobileNet lightweight model were only 4.2 M, and the model size was 13.5 M, which was suitable for embedding in mobile devices and offline operation of embedded devices. Users of basic-level medical institutions can take photos of the anterior segment through the camera so that the device and the local medical computer can be simultaneously diagnosed. Therefore, pterygium screening can be performed at the basic-level hospital. The training images of the MobileNet2 model were obtained from the slit lamp digital microscope, which has a certain gap with the anterior segment images taken by the mobile device camera. In the future, more images of the anterior segment taken by the mobile device camera will be collected to improve the model and make the model more suitable for mobile applications. People in the vast rural areas and remote mountainous areas of China have difficulty seeing a doctor. Mobile devices can realize self-screening, which is convenient for users to pay attention to their ocular surface health at any time.

## 5. Conclusion

This study used deep learning methods to propose a three-category intelligent lightweight-assisted pterygium diagnosis model, MobileNet, based on amplified data. Its results were compared with three classic deep learning models (AlexNet, VGG16, and ResNet18). The MobileNet 2 model had the fewest parameters, and its overall evaluation index results were the best. This model can be used on low-configuration computers at basic-level hospitals, and it can help primary doctors preliminarily screen pterygium problems in patients through anterior segment images. It can also provide suitable recommendations and timely referrals, improve the diagnosis level of primary ophthalmology, and obtain good social benefits. Additionally, the small size and few parameters of the lightweight model lay the foundation for future models to be embedded in mobile devices so that it is convenient for mobile users to screen themselves for pterygium problems.

## Figures and Tables

**Table 1:** Diagnostic results of MobileNet 1 (original data).

Clinical	MobileNet diagnosis (original data)			
	Normal	Observe	Surgery	Total
Normal	59	2	0	61
Observe	4	45	13	62
Surgery	1	8	56	65
Total	64	55	69	188

**Table 2:** Diagnostic results of MobileNet 1 (augmented data).

Clinical	MobileNet diagnosis (augmented data)			
	Normal	Observe	Surgery	Total
Normal	59	2	0	61
Observe	2	52	8	62
Surgery	0	10	55	65
Total	61	64	63	188

**Table 3:** The five models' evaluation results.

Model	Evaluation indicators	Normal	Observe	Surgery
MobileNet (original data)	Sensitivity	96.72%	72.58%	86.15%
	Specificity	96.06%	92.06%	89.43%
	F1-score	94.40%	76.92%	83.58%
	AUC	0.964	0.823	0.878
	95% CI	0.931-0.996	0.751-0.895	0.820-0.936
	Kappa	77.64%		
	Accuracy	85.11%		
	Size (MB)	13.5		
	Parameters (million)	4.2		
	Time-S (ms)	5.86		
	Time-C (ms)	473.37		
MobileNet (augmented data)	Sensitivity	96.72%	83.87%	84.62%
	Specificity	98.43%	90.48%	93.50%
	F1-score	96.72%	82.54%	85.94%
	AUC	0.976	0.872	0.891
	95% CI	0.947-1	0.811-0.933	0.833-0.948
	Kappa	82.44%		
	Accuracy	88.30%		
	Size (MB)	13.5		
	Parameters (million)	4.2		
	Time-S (ms)	5.75		
	Time-C (ms)	465.53		
AlexNet	Sensitivity	91.80%	83.87%	84.62%
	Specificity	98.43%	88.10%	77.61%
	F1-score	94.12%	80.62%	85.94%
	AUC	0.951	0.860	0.891
	95% CI	0.909-0.993	0.797-0.922	0.833-0.948
	Kappa	80.05%		
	Accuracy	86.70%		
	Size (MB)	233		
	Parameters (million)	60		
	Time-S (ms)	1.06		
	Time-C (ms)	64.63		
VGG16	Sensitivity	96.72%	79.03%	67.69%
	Specificity	92.13%	81.75%	97.56%
	F1-score	90.77%	73.13%	78.57%
	AUC	0.944	0.804	0.826
	95% CI	0.907-0.982	0.733-0.874	0.754-0.899
	Kappa	71.34%		
	Accuracy	80.85%		
	Size (MB)	527		
	Parameters (million)	138		
	Time-S (ms)	1.72		
	Time-C (ms)	1020.11		
ResNet18	Sensitivity	81.97%	66.13%	75.38%
	Specificity	95.28%	81.75%	84.55%
	F1-score	85.47%	65.08%	73.68%
	AUC	0.886	0.739	0.800
	95% CI	0.825-0.947	0.660-0.819	0.728-0.871
	Kappa	61.67%		
	Accuracy	74.47%		
	Size (MB)	44.6		
	Parameters (million)	33		
	Time-S (ms)	2.53		
	Time-C (ms)	170.88		

## Data Availability

The datasets used and/or analysed during the present study are available from the corresponding author on reasonable request.
